# *Entamoeba histolytica* Up-Regulates MicroRNA-643 to Promote Apoptosis by Targeting XIAP in Human Epithelial Colon Cells

**DOI:** 10.3389/fcimb.2018.00437

**Published:** 2019-01-08

**Authors:** Itzel López-Rosas, César López-Camarillo, Yarely M. Salinas-Vera, Olga N. Hernández-de la Cruz, Carlos Palma-Flores, Bibiana Chávez-Munguía, Osbaldo Resendis-Antonio, Nancy Guillen, Carlos Pérez-Plasencia, María Elizbeth Álvarez-Sánchez, Esther Ramírez-Moreno, Laurence A. Marchat

**Affiliations:** ^1^Catedrática CONACYT, Laboratorio de Genómica Funcional y Biología Molecular, Colegio de Postgraduados Campus Campeche, Campeche, Mexico; ^2^Posgrado en Ciencias Genómicas, Universidad Autónoma de la Ciudad de Mexico, Mexico City, Mexico; ^3^Catedrático CONACYT, Instituto Politécnico Nacional, Mexico City, Mexico; ^4^Departamento de Infectómica y Patogénesis Molecular, Centro de Investigación y de Estudios Avanzados del Instituto Politécnico Nacional, Mexico City, Mexico; ^5^Instituto Nacional de Medicina Genómica y Coordinación de la Investigación Científica, Red de Apoyo a la Investigación, Universidad Nacional Autónoma de Mexico, Mexico City, Mexico; ^6^Unidad de Análisis Cuantitativo de Imágenes, Instituto Pasteur, Paris, France; ^7^Unidad de Biomedicina, Facultad de Estudios Superiores-Iztacala, Universidad Nacional Autónoma de México, Mexico City, Mexico; ^8^Instituto Nacional de Cancerología, Mexico City, Mexico; ^9^Programa en Biomedicina Molecular y Red de Biotecnología, Escuela Nacional de Medicina y Homeopatía, Instituto Politécnico Nacional, Mexico City, Mexico

**Keywords:** *Entamoeba histolytica*, apoptosis, microRNAs, SW480, XIAP

## Abstract

MicroRNAs (miRNAs) are small non-coding RNAs that function as negative regulators of gene expression. Recent evidences suggested that host cells miRNAs are involved in the progression of infectious diseases, but its role in amoebiasis remains largely unknown. Here, we reported an unexplored role for miRNAs of human epithelial colon cells during the apoptosis induced by *Entamoeba histolytica*. We demonstrated for the first time that SW-480 colon cells change their miRNAs profile in response to parasite exposure. Our data showed that virulent *E. histolytica* trophozoites induced apoptosis of SW-480 colon cells after 45 min interaction, which was associated to caspases-3 and -9 activation. Comprehensive profiling of 667 miRNAs using Taqman Low-Density Arrays showed that 6 and 15 miRNAs were significantly (*FC* > 1.5; *p* < 0.05) modulated in SW-480 cells after 45 and 75 min interaction with parasites, respectively. Remarkably, no significant regulation of the 6-miRNAs signature (miR-526b-5p, miR-150, miR-643, miR-615-5p, miR-525, and miR-409-3p) was found when SW-480 cells were exposed to non-virulent *Entamoeba dispar*. Moreover, we confirmed that miR-150, miR-643, miR-615-5p, and miR-525 exhibited similar regulation in SW-480 and Caco2 colon cells after 45 min interaction with trophozoites. Exhaustive bioinformatic analysis of the six-miRNAs signature revealed intricate miRNAs-mRNAs co-regulation networks in which the anti-apoptotic XIAP, API5, BCL2, and AKT1 genes were the major targets of the set of six-miRNAs. Of these, we focused in the study of functional relationships between miR-643, upregulated at 45 min interaction, and its predicted target X-linked inhibitor of apoptosis protein (XIAP). Interestingly, interplay of amoeba with SW-480 cells resulted in downregulation of XIAP consistent with apoptosis activation. More importantly, loss of function studies using antagomiRs showed that forced inhibition of miR-643 leads to restoration of XIAP levels and suppression of both apoptosis and caspases-3 and -9 activation. Congruently, mechanistic studies using luciferase reporter assays confirmed that miR-643 exerts a postranscripcional negative regulation of XIAP by targeting its 3′-UTR indicating that it's a downstream effector. In summary, we provide novel lines of evidence suggesting that early-branched eukaryote *E. histolytica* may promote apoptosis of human colon cells by modulating, in part, the host microRNome which highlight an unexpected role for miRNA-643/XIAP axis in the host cellular response to parasites infection.

## Introduction

*Entamoeba histolytica* is the single-celled protozoan parasite causative of human amoebiasis that affects between 40 and 50 million people worldwide. About 10% of infected individuals are at risk for developing invasive amoebiasis, namely amoebic colitis and extra-intestinal disease, such as amoebic liver abscesses that can be fatal (Stanley, 2003). The parasite infection shown clinical variability associated to intestinal microbiota composition that may increase resistance to infection by decreasing the virulence properties and altering systemic immunity against parasites (Burgess et al., 2017). Indeed, specific gut microbiota patterns have been linked to colonization with parasitic protists. For instance, it was reported a differential fecal microbiota in subjects infected with *Giardia duodenalis* or *Entamoeba spp./Blastocystis hominis* (Iebba et al., 2016). Another study found that the *Entamoeba* is significantly correlated with microbiome composition and diversity, and that colonization can be predicted with 79% accuracy based on the composition of an individual's gut microbiota (Morton et al., 2015). Gilchrist et al. also reported that a high parasite burden coupled with increased levels of Prevotella copri was linked to symptomatic infection with *E. histolytica* in children (Gilchrist et al., 2016). In addition, dysbiosis induced by antibiotic treatment increased the severity of amebic colitis and delayed clearance of *E. histolytica* in an amoebic colitis mouse model (Watanabe et al., 2017). These data urge for a better understanding of the mechanisms underlying microbiota-mediated protection that may help explain clinical variability and help treat amoebiasis.

The main site of *E. histolytica* infection is the colon epithelium. Tissues damage resulting from adhesion, lysis, and phagocytosis of host cells is caused by the activity of several parasite proteins; however, the molecular mechanisms by which trophozoites cause epithelial damage are not fully understood. The activity of several parasite proteins including cysteine proteases (Sajid and McKerrow, 2002), the Gal/GalNAc lectin (Petri and Schnaar, 1995), and amoebapores (Leippe, 1997) among others, is important for disruption and invasion of colonic mucosa by *E. histolytica* trophozoites. Moreover, adherence of virulent amoebae to host cells results in cell death, mainly by apoptosis, both *in vitro* (Berninghausen and Leippe, 1997; Sim et al., 2007) and *in vivo* (Moncada et al., 2006), as well as in tissue inflammatory response (Seydel et al., 1997, 1998; Seydel and Stanley, 1998). These events are the result of the ability of parasites to alter gene expression in host cells. Several reports confirmed these assumptions, for instance genome-wide transcriptional analyses of mouse liver cells revealed the impact of *E. histolytica* on transcription of infected cells which contributes to the activation of apoptosis, regenerative and inflammatory cellular pathways in host cells (Pelosof et al., 2006). Also, transcriptional response to adhesion of virulent parasites to liver sinusoidal endothelial cells leads to death and actin cytoskeleton disorganization of host cells (Faust et al., 2011). These data highlights the impact of *E. histolytica* on the gene expression programs of human cells during infection.

Over the last decade, microRNAs (miRNAs) have emerged as a new prominent class of negative regulators of gene expression. MiRNAs are evolutionary conserved small non-coding single-stranded RNAs of 21–25 nt length which function as guide molecules in posttranscriptional gene silencing by binding to the 3′ untranslated region (3′UTR) of target genes resulting in mRNA degradation or translational repression in P-bodies (Bartel, 2004). Notably, aberrant expression of microRNAs may greatly contribute to development of diverse infectious diseases. Interestingly, miRNAs have been investigated in the host-pathogen interactions including viral, bacterial, fungus, and parasitic infections where they mainly mediate inflammatory response and apoptosis in response to inflection (Drury et al., 2017). For instance, *Toxoplasma gondii* inhibits the apoptotic response of infected host cells through upregulation of miR-17-92 expression and downregulation of pro-apoptotic Bim in human macrophages challenged with parasites (Cai et al., 2014). In addition, infection of cholangiocytes with *Cryptosporidium parvum*, a protist causing intestinal and biliary disease, decreased the expression of primary let-7i and mature let-7 which in turn regulate TLR4 expression and contributes to epithelial immune responses against parasite infection (Chen et al., 2007). These findings suggested that miRNAs mediate post-transcriptional regulation of cellular pathways critical to host-cell regulatory responses to parasite infection. Importantly, miRNAs may also represent *bonna fide* biomarkers of infections, mainly in virus and bacterial infections, as its levels significantly differs in patients relative to healthy individuals. For instance, it was reported that miR-18a, miR-21, miR-29, miR-106b, and miR-122 were downregulated in serum of patients with Hepatitis B virus infection and liver cirrhosis relative to patients with chronic hepatitis B without liver cirrhosis. This set of miRNAs was able to distinguish both groups of patient's sensitivity of 85 and specificity of 70% (Jin et al., 2015). These data highlighted the potential of miRNAs as novel diagnostic tools in infectious diseases. However, no studies on miRNAs as potential amoebiasis biomarkers, neither clinical data about the relevance of miRNAs in outcome of patients with intestinal diseases have been reported yet. One mechanism by which pathogens may survive and disseminate in host tissues is by inducing cell death and apoptosis of cells. The effects of parasites on host miRNAs expression have been described in a few protozoan and nematode species, but nothing is known in *E. histolytica*. Moreover, the exact role of miRNAs in cell death of host cells is largely unknown in the context of amoebiasis. In this study, we aimed to determine whether the modulation of host cellular miRNAs is involved in the pathophysiological responses of epithelial colon cells to amoeba interactions. Our results revealed for the first time a miRNAs signature of human SW-480 and Caco2 epithelial colon cells in response to exposure to virulent *E. histolytica* trophozoites. Also, we showed a functional role for miR-643/XIAP axis in the apoptosis activation of host cells.

## Materials and Methods

### Parasite and Cell Cultures

Virulent *E. histolytica* trophozoites (strain HM1:IMSS) were grown under axenic conditions in Diamond's TYI-S-33 medium at 37°C (Diamond et al., 1978). Trophozoites in exponential phase of growth were used in all experiments. SW-480 (CCL-228) and Caco2 (HTB-37) human colorectal adenocarcinoma cell lines were purchased from ATCC collection and cultured in Dulbecco's modified Eagle's medium (DMEM; Invitrogen), supplemented with 10% fetal bovine serum, 100 U/ml penicillin and 100 mg/ml streptomycin. Cultures were maintained in a 5% CO_2_ humidified atmosphere at 37°C. For interaction assays, SW-480 and Caco2 cells (250,000) were cultured overnight in 6-well plates until reach 100% confluence, then amoeba was incubated with monolayers at 10:1 ratio (SW-480:amoeba) during 0, 15, 30, 45 and 75 min for downstream analysis.

### Transmission Electron Microscopy

After 15, 30, and 45 min interactions, SW-480/trophozoites co-cultures were washed twice with PBS to remove any unattached amoeba and fixed for 60 min with 2.5 % (v/v) glutaraldehyde in 0.1 M sodium cacodylate buffer, pH 7.2, and postfixed for 60 min with 1 % (w/v) osmium tetroxide in the same buffer. After dehydration, with increasing concentrations of ethanol and propylene oxide, samples were embedded in Polybed epoxy resins and polymerized at 60°C for 24 h. Thin sections (60 nm) were obtained and stained with uranyl acetate and lead citrate for its examination in a Philips Morgagni 268 D electron microscope.

### miRNAs Profiling Using TaqMan Low Density Arrays

Profiling of microRNAs was performed as previously described with minor modifications (Flores-Pérez et al., 2016). Briefly, Total RNA was extracted from SW-480 cells, with or without interaction with *E. histolytica* trophozoites using the TRIzol reagent (Invitrogen). RNA integrity was assessed using capillary electrophoresis system Agilent 2100 Bioanalyzer with the eukaryotic nano-chip. Only samples with RNA integrity number (RIN) of six or higher were processed. Expression analysis of 667 human miRNAs was performed by reverse transcription and quantitative real-time polymerase chain reaction (RT-qPCR) using the Megaplex TaqMan Low-Density Arrays (TLDAs) v2.0 system (ThermoFisher), as described by the manufacturer. Briefly, 300 ng total RNA were retrotranscribed using stem-loop primers. In order to detect low abundant microRNAs, a pre-amplification step was included. The pre-amplified product was loaded into the TLDA and amplification signal detection was performed in the 7900 FAST real time thermal cycler (ABI). Data were exported to the DataAssist software version 2.0 (Life Technologies) and normalized using the small-nucleolar RNA RNU44. Mean relative quantity (RQ) was calculated and microRNAs differentially expressed between groups (SW-480 cells interacting with trophozoites vs. SW-480 cells alone) were defined as those with fold change >1.5- and *p-*value < 0.05.

### Quantitative Real-Time Reverse Transcription-PCR (qRT-PCR)

The differential expression of six selected miRNAs (hsa-miR-409-3p, hsa-miR-526b-5p, hsa-miR-643, hsa-miR-150, hsa-miR-615-5p and hsa-miR-525) was verified using microRNA assays (Applied Biosystems, Foster City, CA, USA). Briefly, total RNA from SW-480 cells with and without interaction with *E. histolytica*, was reverse transcribed using a specific stem-loop RT primer for each miRNA and the MultiScribe™ reverse transcriptase. Then, diluted retro-transcription reaction (1:15) was independently mixed with Universal PCR Master Mix, No AmpErase® UNG (2X), in presence of individual PCR probes for miR-409-3p, miR-526b-5p, miR-150, miR-643, miR-615-5p, and miR-525. The PCR reactions were done in a GeneAmp® PCR System 9700 (Applied Biosystems), using the following program: 95°C for 10 min, and 40 cycles of 95°C for 15 s and 60°C for 1 min. The relative expression of microRNAs was measured by qRT-PCR using the comparative Ct (2^−Δ*ΔCT*^) method. The snoRNA RNU44 was used as an internal control for data normalization.

### Prediction of miRNAs Targets

MiRNA targets were predicted by using four microRNA target prediction programs: DIANA-microT (http://diana.imis.athena-innovation.gr/DianaTools/index.php?r=microT_CDS/index) [44], TargetScan (http://genes.mit.edu/targetscan) [45], miRanda (http://www.microrna.org) [46] and PicTar (http://pictar.mdc-berlin.de/). DIANA-microT identifies targets that are conserved in human and mouse; TargetScan reveals targets which are conserved in human, mouse, rat, chicken, and dog. miRanda detects targets which are conserved among human, mouse, and rat; and PicTar finds targets which are conserved in human, chimpanzee, mouse, rat, and dog. We selected all target-genes miRNAs pairs that were predicted by at least three programs. Biological pathways of predicted target-genes microRNAs were identified using mirPath (http://diana.imis.athena-innovation.gr/DianaTools/index.php?r=mirpath/index), miRWalk (http://www.umm.uni-heidelberg.de/apps/zmf/mirwalk/predictedmirnapathway.php) and KEGG (http://www.genome.jp/kegg/pathway.html) databases.

### Apoptosis Assays

Apoptosis of SW-480 cells after 15, 30, 45, and 75 min interaction with *E. histolytica* trophozoites was quantified by flow cytometry. Annexin-V/propidium iodide (PI) double assay was performed using the annexin V-EGFP apoptosis detection kit (Roche). For interaction assays, SW-480 cells (250,000) were cultured overnight in 6-well plates until reach 100% confluence, then amoeba was incubated with monolayers at 10:1 ratio (SW-480:amoeba) during 0, 15, 30, 45, and 75 min. Subsequently, cells were washed twice with cold 1X PBS to detach amoebas. Then monolayers were seeded and resuspended in 400 μl binding buffer and stained with 5 μl annexin V-EGFP according to the manufacturer's recommendations. Then, 10 μl PI were added and cells were incubated for 5 min at 4°C in the dark. Cells were analyzed in a FACS Calibur flow cytometer (BD Biosciences). The SW-480 cells cultured in DMEM or treated with 50 μM cisplatin for 24 h were used as controls. Assay was repeated three times in triplicate and data were expressed as mean ± standard deviation. Statistical analyses were performed using the Student's *t-*test and a *p-*value < 0.05 was set as significant.

### Western Blots Assays

For interaction assays, SW-480 cells (250,000) were cultured overnight in 6-well plates until reach 100% confluence, then amoeba was incubated with monolayers at 10:1 ratio (SW-480:amoeba) during 0, 15, 30, and 45 min. Subsequently, cells were washed twice with cold 1X PBS to detach amoebas. Then remaining SW-480 cells were lysed in ice-cold cell lysis buffer (20 mM Tris, 250 mM NaCl, 2 mM EDTA, 1% Triton X-100, 0.4 mM PMSF), and 1X complete protease inhibitor cocktail (Roche). The protein concentration was determined with the Bradford protein assay kit (BioRad). Total protein extracts (30 μg) were separated through 15% SDS-PAGE and electrotransferred to a nitrocellulose membrane (BioRad) using standard protocols. After blocking with 5% dry skimmed milk, 0.2% Tween-20 in 1X PBS (pH 7.4), membranes were incubated with primary antibodies overnight at 4°C.Then, they were washed twice in 1X PBS with 0.1% Tween-20 for 5 min and incubated for 1 h at room temperature with secondary antibodies conjugated to horseradish peroxidase (HRP) (Jackson). Finally, membranes were washed four times for 10 min in 1X PBS- 0.1% Tween-20 and developed with the ECL Western Blotting substrate (Amersham), according to the manufacturer's instructions. Caspase-3 and caspase-9 antibodies (1:1000 dilution, Cell Signaling) and XIAP antibody (1:3000 dilution, Cell Signaling) were used as primary antibodies. Bands were analyzed by densitometry (myImage Analysis, Thermo), and actin expression was used to normalize data.

### Inhibition of MIR-643 Expression Using Antagomirs

SW-480 cells were cultured in a 24-well plate (40,000 cells/well) and grown until reached a 100% confluence. Then cells were incubated with live *E. histolytica* trophozoites for 45 min. Subsequently, amoebas were removed by repeated washing of SW-480 monolayers with cold 1X PBS. Then, SW-480 cells were transfected with miR-643 inhibitor (at 30 and 50 nM) or scramble sequence (50 nM) using siPORT amine transfection agent (Ambion). Non-transfected SW-480 cells exposed to *E. histolytica* were used as control. After 24 h transfection, SW-480 cells were collected and total RNA was extracted using TRIzol reagent to evaluate miRNA-643 expression by stem-loop qRT-PCR and Western blot assays.

### Luciferase Gene Reporter Assays

A DNA fragment corresponding to the XIAP 3′UTR (which contains a miR-643 biding site) was synthesized and cloned into the *Xba*I site, downstream from the stop codon of the luciferase gene, in the pGL3 vector (GenScript) to obtain the pGL3-XIAP-3′UTR construct. SW-480 cells (40,000 cells/well) were transfected with the pGL3-XIAP-3′UTR plasmid (1 μg) using lipofectamine 2000 (Invitrogen) according to the manufacturer's protocol. A pGL3-SIAH1-3′UTR construct that contains the 3′UTR of SIAH1 gene and lack of a miR-643 biding site was used as control. At 24 h postransfection SW-480 cells were incubated with *E. histolytica* trophozoites for 45 min, and then transfected with anti-miR-643 or scramble as described above. Luciferase activity was measured using the Dual-Luciferase Reporter Assay System (Promega) at 24 h post transfection. Data were normalized against values corresponding to SW-480 cells transfected with pGL3-XIAP without interaction with *E. histolytica*. Each assay was repeated three times in duplicate, and data were expressed as mean ± standard deviation.

### Statistical Analysis

Statistical analyses were performed using the unpaired Student *t*-test to compare each condition with control cells and a p value < 0.05 was considered as significant.

## Results

### *E. histolytica* Alters miRNAs Expression During the Induced Apoptosis of Human SW-480 Colon Cells

**Figure 1:**
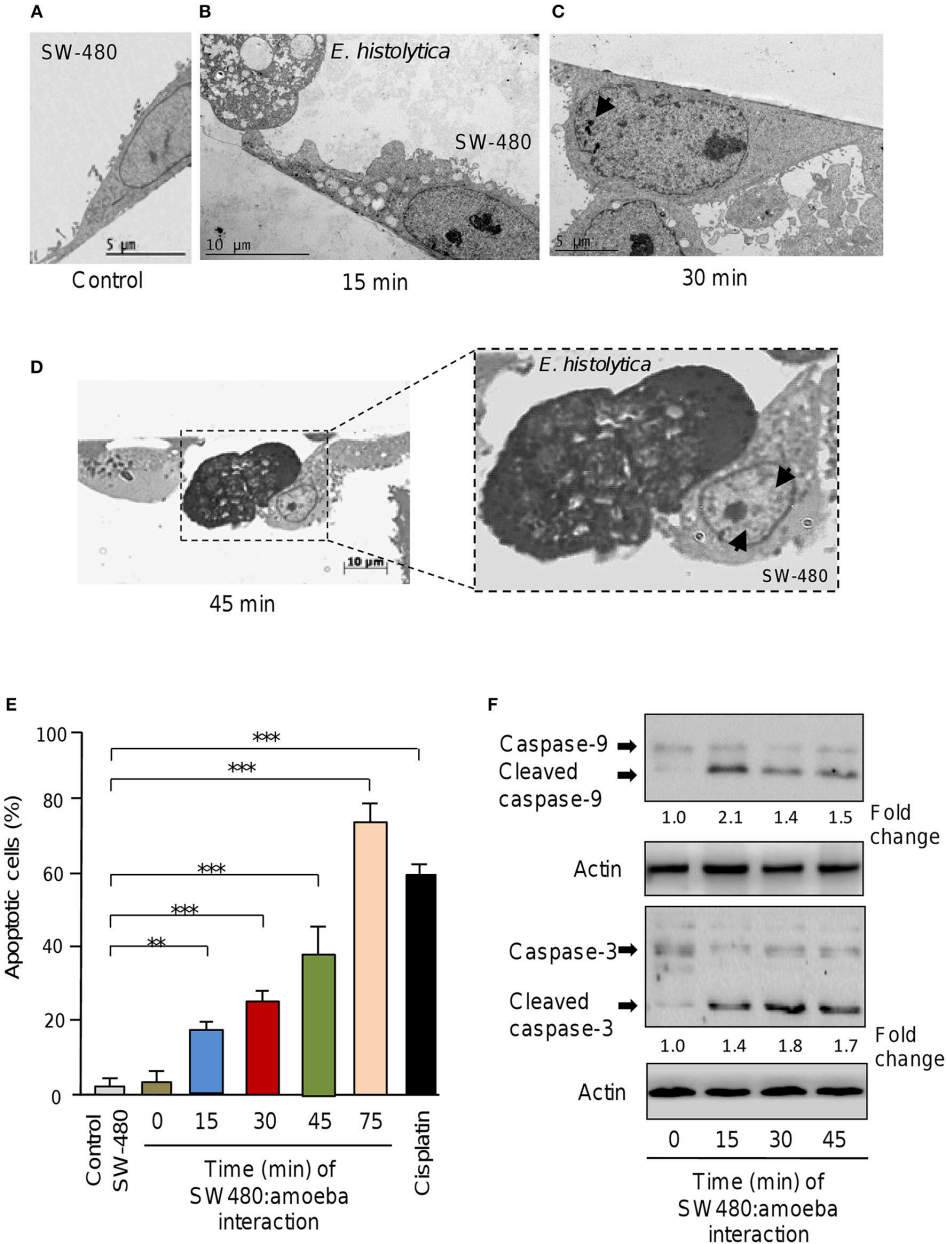
*E. histolytica* induces apoptosis in SW-480 cells. **(A)** Representative transmission electron microscopy ultrastructure image of confluent SW-480 cells showing a well-spread cell with irregular microvilli, mitochondria distributed through the cytoplasm and enlarged nuclei. Scale bar 5 μM. **(B)** Image shows transmission electron microscopy ultrastructure images of trophozoites in contact with colon cells after 15, **(C)** 30 and **(D)** 45 min of co-culture. **(D)** SW-480 cells showing typical early changes associated with apoptosis including a general cell-shrinkage, loss of microvilli and nuclear chromatin condensation denoted by arrows (close up in **D**). Scale bar 10 μM. **(E)** Apoptosis assays. SW-480 cells after interaction with *E. histolytica* trophozoites for 0, 15, 30, 45, and 75 min were analyzed by flow cytometry using annexin V. SW-480 cells without exposure to trophozoites and cells treated with cisplatin (50 μM) for 24 h were used as controls. Data are representative of three independent experiments, performed in triplicate. Bars represent the mean of three independent experiments ± S.D. **p < 0.01. ***p < 0.001. **(F)** Western blot assays showing cropped images for immunodetection of caspase-9 and−3. Whole protein extracts were obtained from SW-480 cells after interaction with trophozoites for 0, 15, 30, and 45 min and submitted to Western blot assays using caspase-3 and caspase-9 antibodies. Actin antibodies were used as loading control. Immunodetected bands intensity was quantified by densitometry and normalized against actin. Numbers between panels represent the fold change values relative to control (time 0).

Previous studies using *in vitro* and *in vivo* approaches demonstrated that *E. histolytica* induced apoptosis of human Caco2 and Jurkat cells (Seydel and Stanley, 1998; Huston et al., 2000, 2003; Kim et al., 2014; Lee et al., 2014). Here we asked about the contribution of non-coding miRNAs in apoptosis of SW-480 epithelial colon cells induced by *E. histolytica* live trophozoites as this issue remains completely understood. Firstly, we setup an interaction model of SW-480 cells with trophozoites and then evaluated the morphological changes induced during the interaction. Data showed that control SW-480 cells incubated without parasites form monolayers of well-spread cells with irregular microvilli (Figure 1A). At early times of interaction between host cells and amoeba (15 min), we hardly observed morphological changes indicative of apoptosis (Figure 1B). In contrast, after 30 and 45 min interaction with trophozoites, the SW-480 cells exhibited the typical early changes associated with apoptosis including a general cell-shrinkage, loss of microvilli, and nuclear chromatin condensation (Figures 1C,B,D). Finally, after 75 min interaction we found extensive SW-480 cell death and monolayers destruction (Supplementary Figures 1, 2) representative of late stages of apoptosis and cell killing, thus we did not considered longer time for downstream molecular analysis. These data indicate that 45 min of amoeba-colon cells interaction was able to induce morphological changes associated with apoptosis in agreement with previous reports in other human cells including colonic Caco-2 and T lymphocyte Jurkat cells (Huston et al., 2000, 2003; Kim et al., 2014; Lee et al., 2014).

To confirm whether *E. histolytica* parasites induce apoptosis in SW-480 cells we performed flow cytometry analysis using annexin V method. Data showed that in the absence of trophozoites a low percentage of SW-480 control cells (3.7 ± 0.4%) were in apoptotic stage. A similar proportion of apoptotic cells (3.9 ± 0.6%) were found at time 0 interaction, which corresponds to initial time when trophozoites and SW-480 cells were co-incubated (Figure 1E). The fraction of apoptotic SW-480 cells significantly increased in a time-dependent way from 19.5% at 15 min co-cultures to 25.1, 40, and 78% at 30, 45, and 75 min interaction, respectively (Figure 1E). In SW-480 cells exposed to apoptosis-inducer cisplatin (50 μM) for 24 h, the proportion of apoptotic cells reached 59%. Next, we were wondering if apoptosis was related to intrinsic pathway activation, therefore we performed Western blot assays for caspase-3 and caspase-9 detection using whole protein extracts from SW-480 cells incubated at different times with virulent trophozoites. Results showed that both caspase-9 and caspase-3 were activated by proteolytic cleavage at 15, 30, and 45 min interaction with amoeba (Figure 1F). In contrast, we did not detected significant amounts of cleaved caspases in control cells (time 0). Actin used as control did not show significant changes during course of time. These data confirmed that *E. histolytica* trophozoites induced apoptosis via the intrinsic pathway in SW-480 colon cells.

MiRNAs are small non-coding RNAs that negatively regulate the expression of target genes involved in cell survival, migration, and apoptosis in diverse eukaryotic organisms (Bartel, 2004). We were asked whether apoptosis of SW-480 cells induced by trophozoites was mediated by changes in expression of miRNAs repertoire. To test this hypothesis, we performed a comprehensive profiling of 667 miRNAs using TaqMan Low Density Arrays (TLDAs) in SW-480 cells interacting or not with virulent parasites for 45 and 75 min. These time points were selected as represents the early and late stages of SW-480 cell destruction by trophozoites. Data showed that 6 miRNAs were differentially expressed (*FC* > 1.5; *p* < 0.05) after 45 min interaction. Particularly, 5 miRNAs denoted as miR-526b-5p, miR-150, miR-643, miR-615-5p, and miR-525 were significantly up-regulated, whereas miR-409-3p was down-regulated (Table 1). At 75 min interaction, we found 16 deregulated miRNAs (3 downregulated and 12 upregulated; Table 1). Intriguingly, none common miRNA was modulated at both times.

**Table 1:** MicroRNAs modulated in human SW-480 epithelial colon cells after 45 and 75 min interaction with virulent *E. histolytica* trophozoites.

**MicroRNAs**	**Fold change (log2 RQ)**	***p*-value**	***locus***
**DOWNREGULATED AFTER 45 MIN INTERACTION**			
hsa-miR-409-3p	−3.39	0.031	14q32.31
**UPREGULATED AFTER 45 MIN INTERACTION**			
hsa-miR-526b-5p	13.48	0.003	19q13.42
hsa-miR-643	3.41	0.019	19q13.41
hsa-miR-150	3.14	0.038	19q13.33
hsa-miR-615-5p	6.97	0.039	12q13.13
hsa-miR-525	8.67	0.044	19q13.42
**DOWNREGULATED AFTER 75 MIN INTERACTION**			
miR-10a	−5.33	0.0344	17q21.32
miR-642	−5.60	0.0487	19q13.32
miR-501	−7.61	0.0156	Xp11.23
**UPREGULATED AFTER 75 MIN INTERACTION**			
miR-100	2.02	0.0281	11q24.1
miR-452	3.02	0.0444	Xq28
miR-135a	3.07	0.0179	3p21.2
miR-26b	3.08	0.0047	2q35
miR-32	4.51	0.0387	9q31.3
miR-502-3p	4.53	0.0334	Xp11.23
miR-652	6.18	0.0220	Xq23
miR-101	4.49	0.0296	1p31.3
miR-221	4.14	0.0041	Xp11.3
miR-505*	4.97	0.0296	Xq27.1
miR-17	5.09	0.0344	13q31.3
miR-656	15.20	0.0334	14q32.31

**Figure 2:**
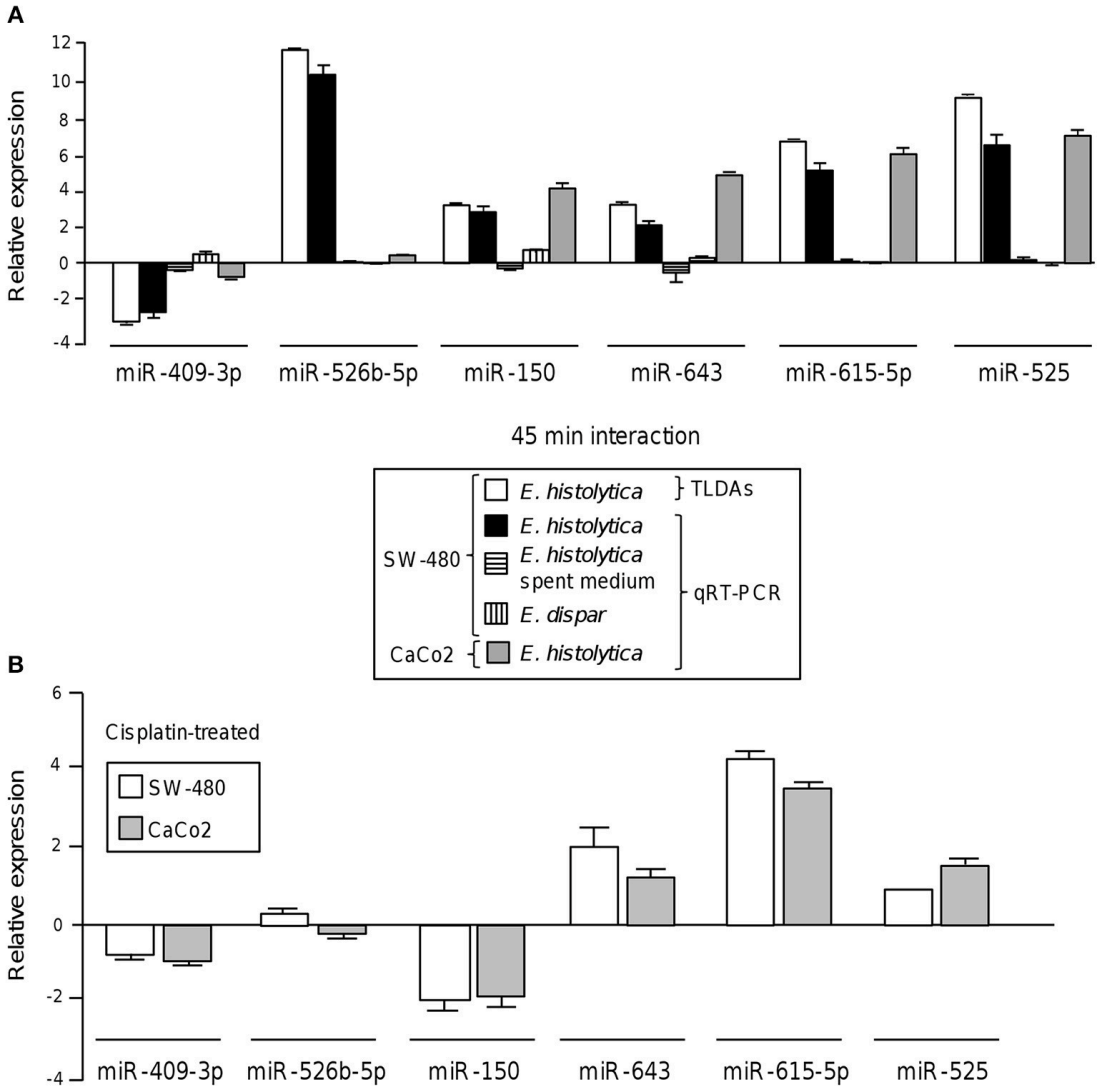
Validation of differential expression of cellular miRNAs after interaction of colon cells with trophozoites. **(A)** Expression levels of the 6-miRNAs signature during the interplay of SW-480 cells with *E. histolytica* for 45 min using qRT-PCR assay (black bars) in comparison with data obtained using spent *E. histolytica* media, during interaction of *Entamoeba dispar* parasites with SW-480 cells and relative to TLDAs profiling (white bars). qRT-PCR assays of Caco2 cells interacting with trophozoites were carried out as an additional control (gray bars). Cell lines, treatments and experiments performed for the 6-miRNAs profiling are denoted in the box inset. **(B)** qRT-PCR assays of the 6-miRNAs in SW-480 and Caco2 colon cells treated with cisplatin as apoptosis control for 24 h. Data is representative of three independent experiments that were performed in duplicate, error bars represent S.D.

To validate these findings, we selected the six-miRNAs signature deregulated at 45 min and reexamined its expression by stem-loop qRT-PCR using individual TaqMan probes. In agreement with TLDAs data, the expression of miR-526b-5p, miR-643, miR-150, miR-615-5p, and miR-525 was found increased, while the abundance of miR-409-3p was decreased in SW-480 colon cells after 45 min interaction with trophozoites (Figure 2A). To distinguish the possibility that a soluble factor released by trophozoites could be contributing in the induction of changes in miRNAs abundance, we carried out qRT-PCR analysis of the six-miRNAs signature in the presence of spent medium obtained from parasite's culture media growing without colon cells and compared with the miRNAs levels found after 45 min contact of trophozoites with SW-480 cells. Data showed that the expression of the six miRNAs was very low in the presence of spent culture media in comparison to incubation with trophozoites, indicating no significant regulation of miRNAs (Figure 2A). In addition, we also ruled out the possibility that virulent-deficient parasites could be inducing changes in the miRNAs abundance. Results showed no significant expression on the six-miRNAs signature after interaction of SW-480 cells with non-virulent *Entamoeba dispar* trophozoites (Figure 2A). We further confirmed the changes of the six-miRNAs levels using an additional Caco2 epithelial colon cell line. Results showed that miR-150, miR-643, miR-615-5p, and miR-525 exhibited similar levels in SW-480 and Caco2 cells after 45 min incubation with virulent trophozoites (Figure 2A). In contrast, miR-409-3p and miR-526b-5p were expressed at very low levels in Caco2 cells in comparison to SW-480 cell line. Then, we analyzed the 6-miRNAs expression in SW-480 and Caco2 cells in the presence of cisplatin used as cell death inductor. Data showed that cisplatin modulated the expression of miR-409-3p, miR-643, miR-615-5p, and miR-525 in SW-480 and Caco2 cells in a similar way as observed after the 45 min interactions with virulent trophozoites (Figure 2B). Moreover, minimal differences in the 4-miRNAs levels in both cell types were observed after cisplatin treatment. In contrast, miR-526b-5p and miR-150 showed an inverse expression profile in cisplatin-treated SW-480 cells in comparison to the observed after SW-480/amoeba interactions without cisplatin.

### Modulated miRNAs Potentially Target Multiple Apoptosis-Related Genes

**Figure 3:**
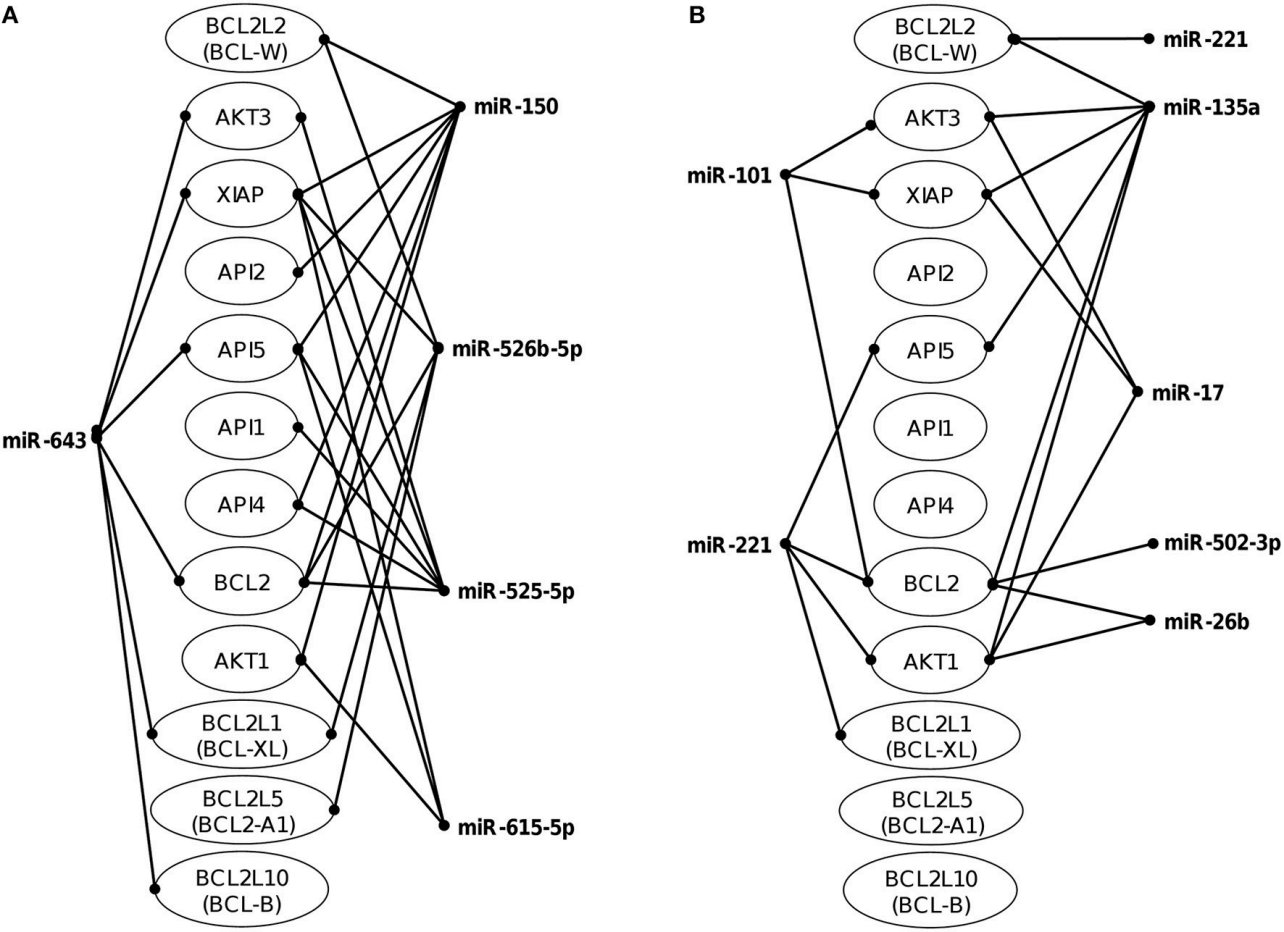
Co-regulation networks of modulated miRNAs and predicted target mRNAs. Image depicts the interaction networks of miRNAs modulated at **(A)** 45 min and **(B)** 75 min after interaction of SW-480 cells with *E. histolytica*. Target mRNAs were predicted by at least two of the three programs used (DIANA-microT, TargetScan, and miRanda). Genes related to apoptosis were also included even if they were targeted by only one miRNA.

The identification of genes that could be targeted by miRNAs is a necessary step to understand its functions in host response to parasites. Several of the regulated miRNAs identified here have been previously described in various biological contexts and human diseases, and some gene targets have been functionally validated (Table 2). However, most of these studies have been performed in cancer cells from diverse types of human malignancies and until we know, there are no reports related to infectious diseases. To gain insights into the biological roles of cellular miRNAs that were modulated in SW-480 cells, we performed computational predictions to identify their gene targets using DIANA-microT, TargetScan, PicTar, and miRanda software's. We only focused in the bioinformatic analysis of potential gene targets of the set of miRNAs deregulated at 45 min as it represent the time in which apoptosis was clearly observed. Data showed that 120 predicted gene targets can be regulated by at least two miRNAs modulated at 45 min. In addition, DIANA-mirPath and miRWalk analyses revealed that 5 cellular pathways were mainly affected. These include mRNA surveillance pathway (16 genes), biosynthesis of unsaturated fatty acids (5 genes), ubiquitin mediated proteolysis (16 genes), PI3K/AKAT signaling pathway (5 genes), and apoptosis (8 genes). As others, here we also showed that incubation of amoebae with host cells resulted in apoptosis, therefore we decided to focus on this cellular process. Interestingly, *in silico* analyses revealed a complex miRNAs-mRNAs coregulation network in which the anti-apoptotic XIAP, API5, BCL2, AKT3, and AKT1 genes were the major targets of deregulated miRNAs at both 45 and 75 min (Figure 3; Table 3).

**Table 2:** Validated targets for miRNAs regulated at 45 min in SW-480 cells after interaction with *E. histolytica* according to miRTarBase (http://mirtarbase.mbc.nctu.edu.tw/php/index.php).

**miRNA**	**Validated target^**a**^**	**Protein name^**b**^**	**Biological context and disease**
**DOWNREGULATED MICRORNAs**			
hsa-miR-409-3p	FGA FGB FGG PHF10 ANG IFNG	Fibrinogen alpha chain Fibrinogen beta chain Fibrinogen gamma chain Phd finger protein 10 Angiogenin, ribonuclease, rnase a family, 5 Interferon, gamma	Cardiovascular disease Cardiovascular disease Cardiovascular disease Gastric cancer Fibrosarcoma Primary immune thrombocytopenia
**UPREGULATED MICRORNAs**			
hsa-miR-526b-5p	TRAF6 SLITRK4 UBE2V2 CREB1 FGD4 PRMT2 CBX5 EIF4E3 RIMKLA SCAMP1	TNF receptor-associated factor 6 SLIT and NTRK-like family, member 4 Ubiquitin-conjugating enzyme E2 variant 2 Camp responsive element binding protein 1 FYVE, rhogef, and PH domain containing 4 Protein arginine methyltransferase 2 Chromobox homolog 5 Eukaryotic translation initiation factor 4E family member 3 Ribosomal modification protein rimk-like family member A Secretory carrier membrane protein 1	Colon cancer, esophageal squamous cell carcinoma, and human lung adenocarcinoma cell Breast carcinomas and brain tumor Human colon carcinoma cells Regulation of gonadotropin biosynthesis Charcot-Marie-Tooth neuropathy Breast cancer Chromodomains characterization in human Tumor suppression Central nervous system function Organellar pH homeostasis
hsa-miR-643	TP53IN1 CSF1 ZEB1 NR3C1 ER-alpha	Tumor protein 53-induced nuclear protein 1 Colony stimulating factor 1 (macrophage) Zinc finger E-box binding homeobox 1 Nuclear receptor subfamily 3, group C, member 1 Estrogen-related receptor alpha	Lymphocytic leukemia Narcolepsy Breast cancer cells Cellular stress Myeloma
hsa-miR-150	MYB EGR2 VEGFA P2RX7 IGF2 CXCR4 ZEB1 NOTCH3 FLT3 EP300 TP53	v-*myb* avian myeloblastosis viral oncogene homolog Early growth response 2 Vascular endothelial growth factor a Purinergic receptor p2x, ligand-gated ion channel, 7 Insulin-like growth factor 2 (somatomedin a) Chemokine (c-x-c motif) receptor 4 Zinc finger e-box binding homeobox 1 Notch 3 Fms-related tyrosine kinase 3 E1a binding protein p300 Tumor protein p53	Lymphocyte development Gastric cancer Vascular diseases Cancer epithelial cells Skeletal myogenesis Ischemic heart disease Esophageal squamous cell carcinoma Thymocyte differentiation Leukemia Gastric cancer Lung cancer cells
hsa-miR-615-5p	IGF2	Insulin-Like Growth Factor 2 (Somatomedin A)	Cutaneous melanoma
hsa-miR-525	DOHH KLK4 E2F1 ERBB-2	Deoxyhypusine hydroxylase Kallikrein-related peptidase Transcription factor E2F1 Receptor tyrosine-protein kinase 2	Prostate cancer Lupus erythematosus Gastric cancer Prostate cancer

a *Gene symbol*.

b *Recommended protein name (UniProtKB)*.

**Table 3:** Modulated microRNAs in SW-480 cells interacting with *E. histolytica* for 45 min and predicted targets associated to apoptosis.

**MicroRNAs**	**Predicted target^**a**^**	**Protein name^**b**^**
**Downregulated MICRORNAS**		
miR-409-3p	BCL2 BCL2L11 BCL2L15	B-cell CLL/lymphoma 2 BCL2-like 11 (apoptosis facilitator) BCL2-like 15 (apoptosis facilitator)
**Upregulated MICRORNAS**		
miR-526b-5p	XIAP BAK1 BNIP3L	X-linked inhibitor of apoptosis BCL2-antagonist/killer 1 BCL2/adenovirus E1B 19kDa interacting protein 3-like
miR-643	BCL2L1 BCL2L10 BCL2L11 BCL2L14 XIAP API5	BCL2-like 1 BCL2-like 10 BCL2-like 10 BCL2-like 14 X-linked inhibitor of apoptosis Apoptosis inhibitor 5
miR-150	BCL2L2 NAIF1 CASP7 CASP8 CFLAR AIFM3 AIFM2	BCL2-like 2 Nuclear apoptosis inducing factor 1 Caspase 7, apoptosis-related cysteine peptidase Caspase 8, apoptosis-related cysteine peptidase CASP8 and FADD-like apoptosis regulator Apoptosis-inducing factor, mitochondrion-associated, 3 Apoptosis-inducing factor, mitochondrion-associated, 2
miR-615-5p	BCL2L1 BCLAF1 BMF AIFM3 AATK	BCL2-like 1 BCL2-associated transcription factor 1 Bcl2 modifying factor Apoptosis-inducing factor, mitochondrion-associated, 3 Apoptosis-associated tyrosine kinase
miR-525	BCL2L1 BCL2L13 BAG3 BAG1 XIAP CASP7 BMF	BCL2-like 1 BCL2-like 13 BCL2-associated athanogene 3 BCL2-associated athanogene X-linked inhibitor of apoptosis caspase 7, apoptosis-related cysteine peptidase Bcl2 modifying factor

a *Gene symbol*,

b *Recommended protein name (UniProtKB)*.

### Apoptosis Inhibitor XIAP Is a Target of miR-643

**Figure 4:**
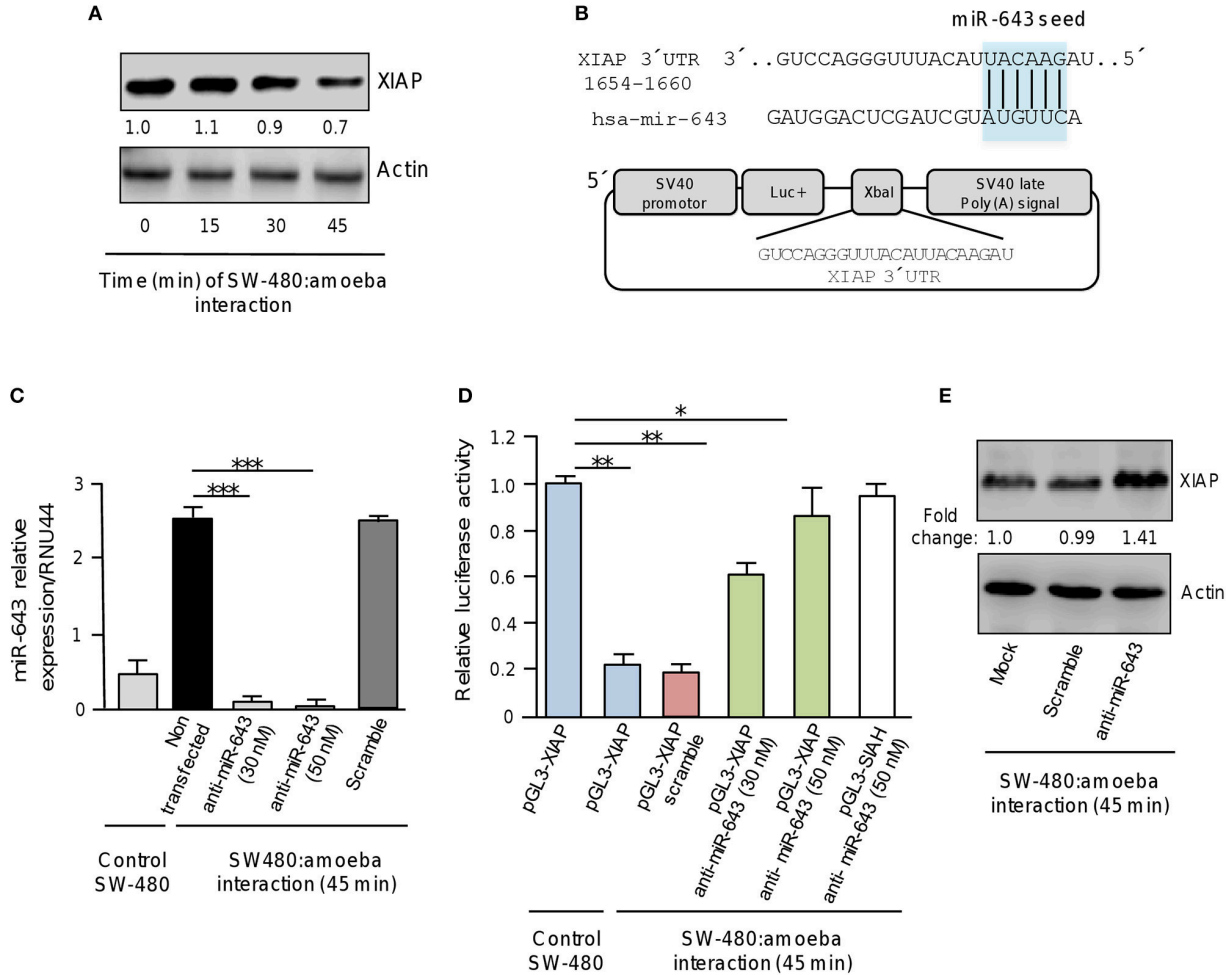
XIAP 3′UTR is a target of miR-643. **(A)** Western blot assays for XIAP expression. SW-480 cells were incubated with trophozoites for 0, 15, 30, and 45 min. Total protein were extracted and XIAP expression was determined by Western blot assays using XIAP antibody (Cell signaling). Actin antibodies (Santa Cruz) were used as control. Numbers represents the fold change in XIAP expression quantified by densitometry and normalized against actin. Values are expressed relative to the control group (time 0 h). **(B)** Schematic representation of pGL3-XIAP construct containing the 3′UTR of XIAP gene cloned at the *Xba*I site downstream of firefly luciferase gene (Luc+) of pGL3 Luciferase Reporter Vector. The miR-643 seed sequence is indicated in the colored box. **(C)** qRT-PCR assays for expression analysis of miR643. SW-480 cells were incubated with trophozoites for 45 min and transfected with miR-643 inhibitor at 30 nM and 50 nM or with scramble sequence (50 nM). **(D)** Luciferase reporter gene assays. SW-480 cells transfected with the pGL3-XIAP were incubated with trophozoites for 45 min and co-transfected with anti-miR-643 (30 and 50 nm) or scramble (50 nM). Luciferase activity was measured after 24 h. SW-480 cells transfected with pGL3-XIAP but not exposed to parasites were used as control. **(E)** Western blot assays showing cropped images for XIAP expression. SW-480 cells transfected with anti-miR-643, scramble or not transfected were incubated with trophozoites for 45 min and then submitted to immunoblotting with XIAP and actin antibodies. Bands intensity was quantified by densitometry and normalized to actin. In all cases, data are representative of three independent experiments by duplicate. Error bars represent S.D. The unpaired Student *t*-test was used to compare each condition with control cells that were not exposed to *E. histolytica*. *p < 0.05; **p < 0.01; ***p < 0.001.

To understand the biological role of cellular miRNAs in apoptosis during *E. histolytica* interplay, we initiated the functional characterization of miR-643 which was upregulated after 45 min interaction of trophozoites with SW-480 cells as it was predicted to modulate the expression of six genes related with apoptosis (Table 1, Figure 3). Bioinformatic analysis indicated that X-linked inhibitor of apoptosis protein XIAP (also known as inhibitor of apoptosis protein 3, IAP3 and baculoviral IAP repeat-containing protein 4, BIRC), a member of the inhibitor of apoptosis family of proteins (IAPs) (Deveraux et al., 1997), it's a predicted target of miR-643. To demonstrate the role of miR-643/XIAP axis in apoptosis, we first evaluated whether interaction of trophozoites with SW-480 cells have a negative effect on expression of XIAP. Western blot results showed that the amount of XIAP protein in SW-480 cells was decreased in a time-dependent way in response to exposition with trophozoites. At 30 and 45 min we found a 10 and 30% reduction in XIAP levels, respectively, in comparison to time 0 (Figure 4A). Actin used as control did not show significant changes during course of time.

Examination of the XIAP gene sequence allowed us to identify a miR-643 binding site at the 3′ UTR (1654 to 1660 nucleotide position) suggesting that it may be a direct target (Figure 4B). Thus to explore whether miR-643 may negatively regulate XIAP, we inhibited it's expression using a specific antagomiR. SW-480 cells were or not exposed to trophozoites for 45 min and transfected with miR-643 inhibitor (30 and 50 nM) or with scramble sequence (50 nM) as negative control. Results from qRT-PCR assays indicated that in control SW-480 cells grown without trophozoites, the relative expression of miR-643 was low (Figure 4C). Interestingly, miR-643 expression was induced six-fold in non-transfected SW-480 cells after interaction with trophozoites in agreement with original TLDAs data (Table 1; Figure 4C). Moreover, we found that miR-643 amount was dramatically reduced in cells transfected with anti-miR-643 when compared with non-transfected and scramble-transfected SW-480 control cells interacting with *E. histolytica*, indicating that both antagomiR doses (30 and 50 nM) were effective to suppress the expression of endogenous miR-643 (Figure 4C).

To corroborate whether miR-643 can exerts posttranscriptional repression of XIAP, we performed luciferase reporter gene assays. A DNA fragment corresponding to the 3′UTR sequence of XIAP which contains the putative miR-643 binding site was cloned downstream of the luciferase coding region into pGL3 vector (Figure 4B). Recombinant pGL3-XIAP plasmid was transfected alone or together miR-643 inhibitor into SW-480 cells and luciferase activity was analyzed after 24 h. Data showed that SW-480 cells transfected with pGL3-XIAP construct alone showed a high luciferase activity which was taken as 100% (Figure 4D). Interestingly, transfection of pGL3-XIAP into SW-480 cells co-incubated with trophozoites for 45 min resulted in a substantial reduction of the relative luciferase activity in comparison with SW-480 monoculture used as control (Figure 4D). A similar reduction (81%) was observed when cells were co-transfected with scramble control. These data suggested that during interaction of amoeba with colon cells endogenous miR-643 levels were increased allowing the repression of pGL3-XIAP mRNA product and luciferase activity. Remarkably, luciferase enzyme activity was restored when expression of miR-643 was inhibited using two different doses of antagomiR (30 and 50 nM). Importantly, in SW-480 cells transfected with 50 nM, luciferase activity almost reached similar levels that observed in cells that were not exposed with amoebas (Figure 4D). This effect was counteracted when cells were co-transfected with anti-miR-643 and an unrelated pGL3-SIAH1 construct (containing the 3′UTR of SIAH1 gene lacking of miR-643 binding sites) used as control and (Figure 4D). Furthermore, Western blot assays corroborated that expression of endogenous XIAP protein was restored (fold change 1.4 in comparison to control) in SW-480 cells exposed to trophozoites when miR-643 was inhibited (Figure 4E), strengthening the notion that miRNA-643 targets the XIAP gene during the interplay of SW-480:amoeba. Taken altogether, these data suggested that XIAP is targeted by miR-643 during interaction of *E. histolytica* trophozoites with human colon cells.

### Inhibition of miR-643 Impairs Apoptosis During Interaction of *E. histolytica* With Colon Cells

**Figure 5:**
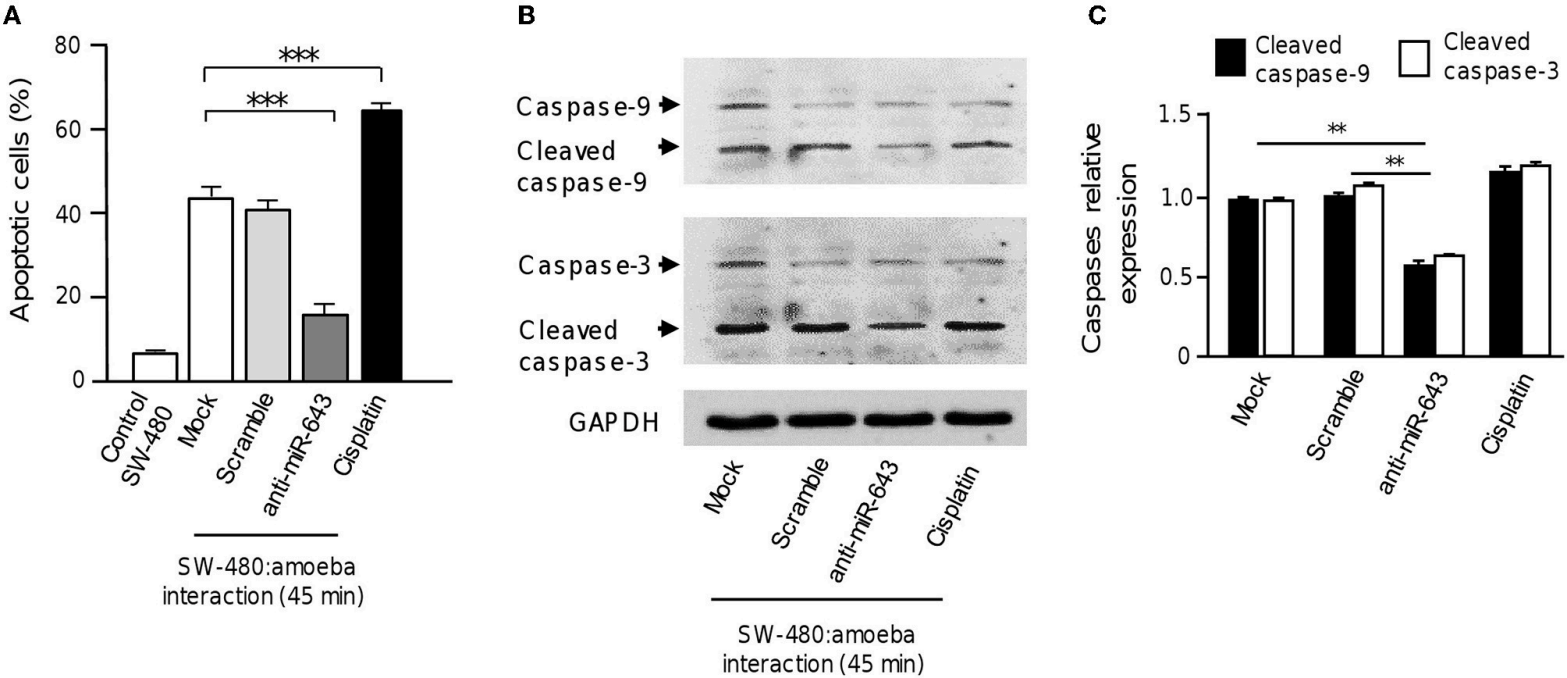
Inhibition of miR-643 impairs apoptosis of SW-480 cells interacting with trophozoites. **(A)** SW-480 cells were or not transfected with miR-643 inhibitor or scramble and interacting with trophozoites for 45 min. Then apoptosis of SW-480 cells was quantified using annexin V assays. SW-480 cells without exposure to *E. histolytica* and cells treated with cisplatin (50 μM, during 12 h) were used as controls. **(B)** Protein extracts from miR-643 deficient SW-480 cells interacting with *E. histolytica* trophozoites for 45 min and controls were submitted to Western blot assays using caspase-3 and caspase-9 antibodies. GADPH antibodies were used as loading control. **(C)** Immunodetected bands intensity in panel B were quantified by densitometry and normalized to GAPDH. Values are expressed relative to the control group (0 h, interaction). **p < 0.01; ***p < 0.001.

To obtain further evidence that upregulation of miR-643 is involved in apoptosis activation during the interplay of parasites with SW-480 cells, the expression of miR-643 was inhibited using antagomiRs and the effects in cell death were quantified using annexin-V assays. Again, results showed that in the absence of trophozoites a low percentage of SW-480 control cells were in apoptosis. In addition, data indicated that the fraction of apoptotic cells significantly decreased to 18% after 45 min interaction of miR-643-deficient SW-480 cells with parasites in comparison to cells transfected with scramble negative control and mock which display around 40% apoptosis (Figure 5A). In SW-480 cells exposed to cisplatin as control the proportion of apoptotic cells reached 61%. Then, we performed Western blot assays for the detection of caspases 3 and 9 using colon cells transfected with anti-miR-643 and controls. Results showed that activation of caspases 3 and 9 was decreased up to 47 and 49%, respectively, after 45 min interaction of antagomiR-643 transfected-SW-480 cells with amoeba (Figures 5B,C) in comparison to controls. Altogether these data suggested that *E. histolytica* induced apoptosis in SW-480 cells, at least in part, through induction of miR-643, which in turn downregulates XIAP.

## Discussion

It has been largely described that *E. histolytica* trophozoites produce important changes in genetic programs of host cells during the invasive process. In addition, the impact of host non-coding RNAs in the fine tuning regulation of genes involved in apoptosis is poorly understood. In the current study, we identified for the first time a specific miRNA-signature of SW-480 colon cells that may be involved in the pathophysiological responses to amoeba infection. Our data revealed that exposure to virulent *E. histolytica* trophozoites resulted in a significant deregulation of miRNAs repertoire in epithelial colon cells, as illustrated by the up-regulation of miR-526b-5p, miR-643, miR-150, miR-615-5p, and miR-525, and the down-regulation of miR-409-3p after 45 min interaction. Of these, 4 miRNAs genes are located at the genomic locus 19q13 that is a chromosomal region frequently altered in many human cancers (Kontos, 2014). The small number of modulated miRNAs suggests that amoeba has a limited, but at the same important, impact on the post-transcriptional control of host gene expression mediated by miRNAs, at least at 45 min interaction. Notably, at longer time of cell-cell interactions (75 min) we found an increased number of modulated miRNAs, but none was common at both times. These intriguingly findings showed and suggested that: (i) the apoptosis process could be differentially regulated by miRNAs at 45 min and 75 min. These data is in agreement with the many differences at morphological and molecular level that we evidenced here using multiple approaches. At 45 min we observed about 40% apoptosis, whereas at 75 min most of colon cells showed apoptosis and presented severe damage and extensive cell death; thus is understood that differences at molecular level (miRNAs profile) also should be exist, (ii) our data also suggested that differential expression of miRNAs is not linear between 45 and 75 min, indicating that at late time of apoptosis a different set of miRNAs is needed to modulate the final stage of cell death, and; (iii) apoptosis is a complex event in which several gene regulation mechanisms are activated/deactivated at different stages of process. The different miRNAs modulated at both times revealed the different stages of apoptosis process and thus it's likely that they change during the time revealing a dynamic regulation of apoptosis by miRNAs. However, additional experimental evidences are needed to demonstrate these assumptions as well to understand the role of the deregulated miRNAs at late stage (75 min) of cell killing.

In related studies, the number of modulated miRNAs in response to parasite infection is variable, depending on the pathogen species and the use of an *in vivo* or *in vitro* model. For example, *Eimeria papillata*, another gastrointestinal protozoan, modulates only 4 miRNAs species in the mouse jejunum (Dkhil et al., 2011), while *Plasmodium, Cryptococus*, and *Leishmania* modulate a set of 19, 28, and 64 microRNAs in hepatocytes, cholangiocytes and fibroblasts, respectively (Chen et al., 2007; Delić et al., 2011; Lemaire et al., 2013). Thus our datasets size is in the range of regulated miRNAs observed in different pathogen-host cells studies. Importantly, one should keep on mind that a single miRNA can potentially regulate as many as 1,000 different genes (Krek et al., 2005). Indeed, the 6-modulated miRNAs at 45 min were individually predicted to regulate from 365 (miR-615) to 1057 (miR-525). Altogether, they can potentially affect the expression of genes participating in at least five important biochemical pathways in SW-480 cells, including mRNA surveillance pathways, biosynthesis of unsaturated fatty acids, ubiquitin mediated proteolysis, PI3K-AKT signaling pathway and apoptosis. These data indicate that trophozoites interaction represents a stress condition that induces a physiological and adaptive response in host cells. The modulation of proteins involved in mRNA surveillance and ubiquitin mediated proteolysis points out that host cells modify their gene expression after *E. histolytica* interaction. Importantly, bioactive lipid molecules promote apoptosis by modulating mitochondrial membrane permeability and activating different enzymes including caspases (Vanhaesebroeck et al., 2010; Huang and Freter, 2015). Amoeba may be inducing apoptosis in host cells by altering, in part, miRNAs regulating genes involved in lipid metabolism, PI3K-AKT signaling pathway and apoptosis. Notably, the six modulated microRNAs potentially target genes related to apoptosis. Thus, the expression of BCL2L11, XIAP, AIFM3, BCL2L1, and BMF can potentially be regulated by at least two of these miRNAs. SW-480 cells were induced to apoptosis and caspase-3 and-9 were processed following interaction with *E. histolytica* trophozoites, suggesting the activation of the apoptotic intrinsic pathway. Several of the miRNAs identified here have been previously described in cancer cells with a role in the regulation of cell proliferation and apoptosis. In addition, a few targets related to apoptosis have been validated. For instance, miR-409 promotes epithelial-to-mesenchymal transition and prostate tumorigenesis (Josson et al., 2015) while it suppresses tumor cell invasion and metastasis by directly targeting radixin in gastric cancer (Zheng et al., 2012). miR-150 promotes cell proliferation in gastric cancer and cancer epithelial cells (Zhou et al., 2008; Wu et al., 2010), while it functions as a tumor suppressor in human colorectal cancer by targeting c-Myb (Feng et al., 2014). miR-526b-5p induces cell cycle arrest and apoptosis in non-small cell lung cancer (Zhang et al., 2015). miR-615 functions as a tumor suppressor in pancreas and breast cancer (Bai et al., 2015; Sun et al., 2015).

**Figure 6:**
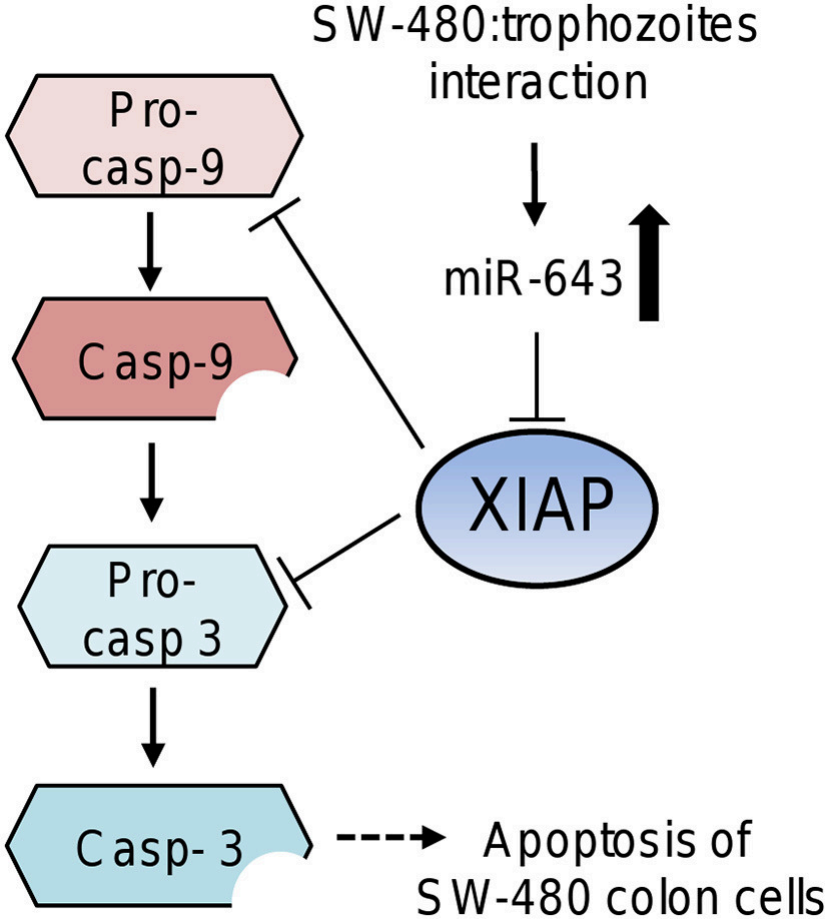
Working model that describes the possible participation of miR-643 in apoptosis of SW-480 cells driven by *E. histolytica*. Upregulation of miR-643 occurs after contact of SW-480 cells with parasites. Then miR-643 may target the XIAP 3′UTR inducing the downregulation of XIAP protein levels, which release the inhibition of caspase-3 and -9. In consequence the executioner caspase-3 triggers degradation of cellular proteins inducing the apoptosis of SW-480 cells.

Here we demonstrated that miR-643 target the anti-apoptotic XIAP gene in SW-480 cells. Previously, it was reported that miR-643 functions as a tumor suppressor gene in osteosarcoma cells. It was showed that miR-643 suppressed the cancer hallmarks and as a *bona fide* tumor suppressor it may also activates apoptosis (Wang et al., 2017). XIAP has a central regulatory role in programmed cell death by inhibiting the caspases cascade (Deveraux and Reed, 1999). In agreement, apoptosis of SW-480 cells was associated with XIAP repression mediated by upregulation of miR-643 in response to *E. histolytica* exposure. It is important to note that miR-643 and the other modulated miRNAs can also potentially regulate the expression of other target genes related to apoptosis, contributing to SW-480 cell death. Based on our results, we proposed a working model to describe the participation of cellular miRNAs in apoptosis driven by amoebas' interaction (Figure 6). In this model, after 45 min contact of SW-480 cells with parasites the miR-643 was upregulated. Then miR-643 target the XIAP 3′UTR inducing the downregulation of XIAP protein levels, which release the inhibition of caspase-3 and -9. In consequence the executioner caspase-3 triggers degradation of cellular components resulting in the apoptosis of SW-480 cells. Notably, miR-526-5p, miR-525, miR-150, and miR-615-5p could also contribute to XIAP inhibition, as they were upregulated during the SW480:amoeba interplay, and were also predicted to target XIAP, however additional experimental evidences are needed to support this assumption.

Limitations of the actual study include: (i) as in previous reports on apoptosis induced by *E. histolytica* in colonic Caco-2 and T lymphocyte Jurkat cells (Huston et al., 2000, 2003; Kim et al., 2014; Lee et al., 2014), the use of a particular cell line limits the extrapolation of findings to *in vivo* conditions; (ii) the miRNAs signature reported here are specific for SW-480 cell line; (iii) the parasite's processes that may trigger miRNAs changes during apoptosis of SW-480 cells remains unknown; (iv) the contribution of additional miRNAs in apoptosis and immune response evasion, and the mechanism by which amoeba contact regulates the transcription of selected host miRNAs remains to be elucidated. Our results showed that the knowledge of the impact of *E. histolytica* on host miRNAs may provide new insights into the relationships between this pathogen and human cells. This first miRNA profiling in SW-480 human colon cells exposed to amoeba revealed significant alterations in cellular miRNAs expression. The set of deregulated miRNAs represent a guide for further functional studies in apoptosis and immune response events. Thus, our findings represent novel data that contribute to our understanding of the cellular and molecular mechanisms activated by the host cells during *E. histolytica* contact.

## Author Contributions

IL-R, YS-V, OH, CP-F, and BC-M conducted all the experiments. BC-M performed the transmission electron microscopy of cells. MÁ-S, ER-M, and LM performed experiments with amoeba. OR-A performed the *in silico* analysis. CL-C, NG, CP-P and LM contributed to experimental design, intellectual input, and interpreting data. CL-C and LM wrote the manuscript.

### Conflict of Interest Statement

The authors declare that the research was conducted in the absence of any commercial or financial relationships that could be construed as a potential conflict of interest.
