# Supplementary figures and images for: Entamoeba histolytica Up-Regulates MicroRNA-643 to Promote Apoptosis by Targeting XIAP in Human Epithelial Colon Cells
Source: Front Cell Infect Microbiol. 2019 Jan 8;8:437. doi: 10.3389/fcimb.2018.00437 (PMC6333105; doi:10.3389/fcimb.2018.00437)

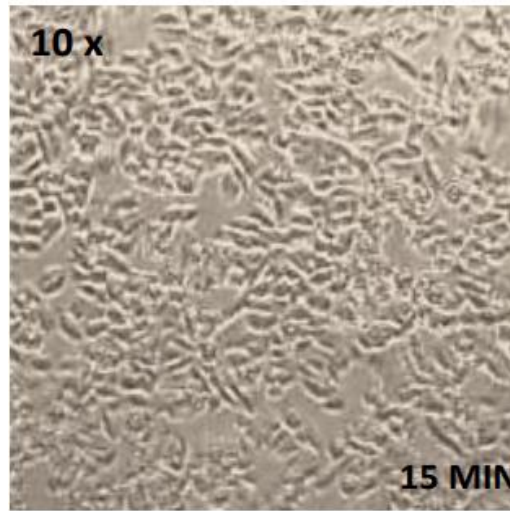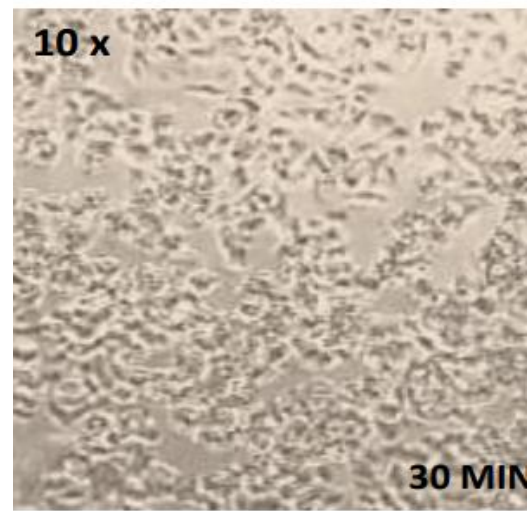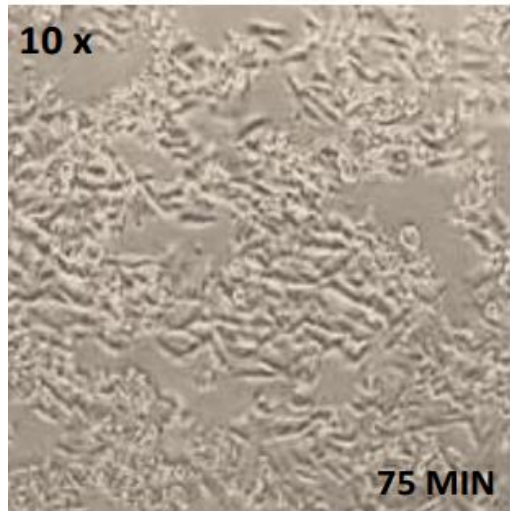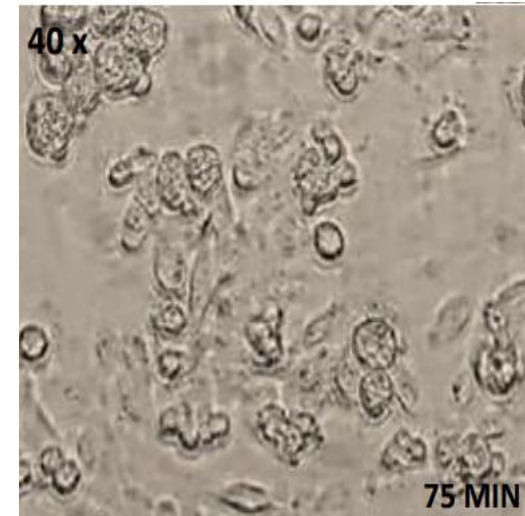

**Supplementary figure 1.** Interaction of SW-480 cells with *E. histolytica* trophozoites (10:1) at 15, 30 and 75 min.

Supplement: Supplementary file 1 [file Data_Sheet_1.PDF]
