# Supplementary material for: Entamoeba histolytica Up-Regulates MicroRNA-643 to Promote Apoptosis by Targeting XIAP in Human Epithelial Colon Cells
Source: Front Cell Infect Microbiol. 2019 Jan 8;8:437. doi: 10.3389/fcimb.2018.00437 (PMC6333105; doi:10.3389/fcimb.2018.00437)
Supplement: Supplementary file 2 [file Data_Sheet_2.PDF]

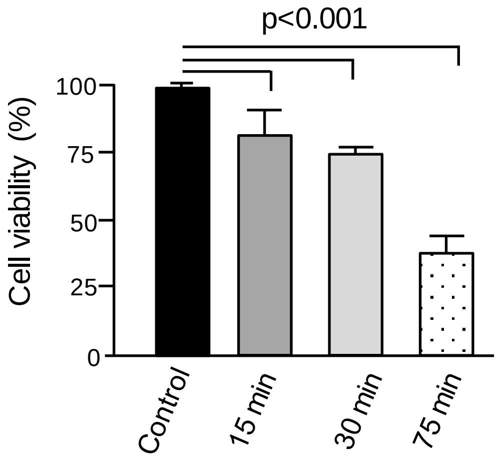

Supplementary figure 2. Cell viability of SW-480 cells after interaction with HMI-IMSS *E. histolytica* virulent trophozoites at time 0 (control), 15, 30 and 75 min measured by MTT assays.
